# Periodontitis and inflammatory bowel disease: a meta-analysis

**DOI:** 10.1186/s12903-020-1053-5

**Published:** 2020-03-12

**Authors:** Yang-yang She, Xiang-bo Kong, Ya-ping Ge, Zhi-yong Liu, Jie-yu Chen, Jing-wei Jiang, Hong-bo Jiang, Si-lian Fang

**Affiliations:** 1 Department of Stomatology, The Sixth Affiliated Hospital, Sun Yat-sen University, No.26 Yuancun Erheng Road, Tianhe District, 510655 Guangzhou, Guangdong People’s Republic of China; 2 Department of Stomatology, Sun Yat-Sen Memorial Hospital, Sun Yat-sen University, No.107 West Yanjiang Road, Yuexiu District, 510120 Guangzhou, Guangdong People’s Republic of China; 3 Visiting Scholar, Department of Periodontics and Oral Medicine, University of Michigan School of Dentistry, Ann Arbor, MI USA; 4 Department of Orthodontics, Guanghua School of Stomatology, Affiliated Stomatological Hospital, Guangdong Province Key Laboratory of Stomatology, Sun Yat-sen University, No.56 West Lingyuan Road, Yuexiu District, 510055 Guangzhou, Guangdong People’s Republic of China; 5 Department of Epidemiology and Biostatistics, School of Public Health, Guangdong Pharmaceutical University, No.283 Jianghai Avenue, Haizhu District, 510310 Guangzhou, Guangdong People’s Republic of China; 6 Visiting Scholar, Section of Oral and Maxillofacial Surgery, Department of Surgery, University of Michigan, Ann Arbor, MI 48109 USA

**Keywords:** Periodontitis, Inflammatory bowel disease, Meta-analysis

## Abstract

**Background:**

Periodontitis was reported to be associated with inflammatory bowel disease (IBD). However, the association between them has not been firmly established in the existing literature. Therefore, this meta-analysis was conducted to evaluate the relationship between periodontitis and IBD.

**Methods:**

Electronic databases were searched for publications up to August 1, 2019 to include all eligible studies. The pooled *odds ratios* (*ORs*) and 95% *confidence intervals* (95% *CIs*) were estimated to determine the association between periodontal disease and IBD using a random or fixed effects model according to heterogeneity.

**Results:**

Six eligible studies involving 599 IBD patients and 448 controls were included. The pooled *OR* between periodontitis and IBD was 3.17 (95% *CI*: 2.09–4.8) with no heterogeneity observed (*I*^*2*^ = 0.00%). The pooled *OR*s were 3.64 (95% *CI*: 2.33–5.67) and 5.37 (95% *CI*: 3.30–8.74) for the associations between periodontitis and the two sub-categories of IBD, Crohn’ s disease and ulcerative colitis, respectively.

**Conclusions:**

The results demonstrated that periodontitis was significantly associated with IBD. However, the mechanisms underlying periodontitis and IBD development are undetermined. Further studies are needed to elucidate this relationship.

## Background

Inflammatory bowel disease (IBD) is a chronic relapsing and remitting intestinal inflammatory disease with an increasing prevalence worldwide [1, 2]. Crohn’ s disease (CD) and ulcerative colitis (UC) are two forms of IBD [3]. While UC is limited to the colon, CD can affect anywhere along the gastrointestinal tract, most frequently in the distal ileum. The main clinical manifestations are abdominal pain, diarrhea and bloody stool. Besides the intestinal inflammatory involvement and complications that characterize the disease, extraintestinal manifestations (EIMs) occur in up to 40% of IBD patients [4], involving the eyes, mouth, nerve system, skin, joints, and liver [5]. Oral lesions precede, coincide with or follow the onset of the intestinal symptoms [6]. However, the prevalence of oral lesions in IBD varied substantially in previous studies [7, 8]. Due to poorly understood etiology, there is currently no cure but only temporary relief for IBD patients [9].

While many potential causes that play a major role in the disease pathogenesis have been identified. These fall into three specific categories: genetic predisposition, the host immune system, as well as environmental factors, such as the gut microbiota dominated by intestinal bacteria [10, 11]. An emerging theory is that IBD is the result of an abnormal reaction of T-lymphocytes to specific bacterial flora in genetically vulnerable populations [12]. Compared with healthy individuals, the composition of intestinal bacterial microbiota of IBD patients were imbalance [13]. The dynamic crosstalk between intestinal epithelial cells (IECs), intestinal microbes and local immune cells reflects one of the essential features of intestinal homeostasis [13].

Earlier studies documented a high prevalence of periodontitis in patients with IBD [14, 15]. However, with regard to the markedly different features between CD and UC [16], information would be lost when they are combined as IBD. Hence, they should be measured separately. Periodontitis is a polymicrobial, biofilm-mediated disease resulting in inflammatory resorption of alveolar bones [17]. Periodontitis and IBD share the inflammatory processes in its progression, in which the key mediators involved in tissue damage are common, such as some cytokines [12, 18]. In addition, a high frequency of periodontopathic bacteria such as *Campylobacter rectus, Porphyromonas gingivalis and Tannerella forsythia* have been found among patients with CD [19]. The periodontal pathogens induced changes of the composition of intestinal microorganisms, and their inflammatory response could cause IECs barrier dysfunction, accentuating the disease [20].

At present, the relationship between periodontitis and IBD has not been firmly established. Confirming this correlation is critical and would inspire future research on understanding IBD etiology, elucidating the underlying mechanisms, and might lead to novel treatment strategies. Therefore, we conducted a meta-analysis on the association between periodontitis and IBD.

## Methods

### Search strategy

In this meta-analysis, we followed the method described in the Meta-Analysis of Observational Studies in Epidemiology guidelines [21]. The following major databases were screened for the following terms: ((((inflammatory bowel disease) OR Crohn’s disease) OR ulcerative colitis)) AND ((((((((periodontal diseases) OR gingival diseases) OR periodontitis) OR gingivitis) OR edentulous) OR edentulism) OR tooth loss) OR teeth loss). English-language publications were extracted from Web of Science, PubMed, Cochrane, and Embase; while Chinese-language articles were retrieved from China National Knowledge Infrastructure (CNKI), Wangfang and CQVIP. The search was limited to literature from before August 1, 2019, including primary researches.

### Study selection

Eligible studies were examined by authors (Yang-yang She and Xiang-bo Kong) independently. Final selection was verified by a third author (Hong-bo Jiang) and disagreements were resolved by discussions. The inclusion criteria of an eligible study were as follow: (1) related to periodontal conditions in patients with IBD; (2) provided at least one of the clinical parameters: bleeding on probing (BOP), clinical attachment loss (CAL), oral plaque index (PI), gingival index (GI), gingival recession (GR), probing pocket depth (PPD), papilla bleeding index (PBI); (3) reported original data; (4) presented cross-sectional studies, cohort studies or case-control studies; (5) Full text in English or Chinese. Case reports, case series, in vitro studies, reviews, abstracts, editorials, and letters were excluded from the selection. In case where multiple publications were based on the same population, the more recent or complete report were considered.

### Data extraction

Data extraction conducted by Yang-yang She and Xiang-bo Kong was based on a standardized, pre-piloted data extraction form. The extracted information included: (1) last name of the first author, publication year, study location, study design and matched variables, (2) study participant demographics including male to female ratio, mean age, total number of IBD, CD, UC cases and controls, (3) periodontal measures including prevalence of periodontitis and risk estimate, BOP, CAL, GI, GR, PI, PPD, and PBI, (4) pharmacological treatments, (5) smoking status of the IBD patients and controls, (6) adjusted variables.

### Quality assessment

Newcastle-Ottawa Scale (NOS) was employed to evaluate the methodological quality of the included studies [22]. Studies with at least five scores were defined as moderate or high methodological quality.

### Statistical methods

The estimates (or adjusted estimates if applicable) and the corresponding 95% *confidence interval* (*CI*) between IBD and periodontitis were used to calculate the pooled estimates. If no estimates were available in the studies, the numbers of IBD cases (with periodontitis or not) and controls (with periodontitis or not) were used to calculate the pooled estimates. Forest plots were performed to assess the individual and pooled estimates with the corresponding 95% *CI*. The random effect model would be applied if the Cochrane *Q* statistic with a significant level of *P* < 0.10 or *I*^*2*^ > 50% [23]. The statistical analysis was accomplished with Stata Version 11.0 (Stata Corp, College Station Texas). The *P* values from the two-tailed tests are statistically significant when *P* < 0.05, except where otherwise specified.

Begg’s test and Egger’s test were applied to test potential publication bias [24]. Funnel plot was generated to assess publication bias by the visual inspection of asymmetry. The *P* values for Begg’s test and Egger’s test less than 0.05 revealed that publication bias existed.

To examine the impact of a single estimate on the pooled estimates, we performed sensitivity analysis by systematically removing one study at a time and recalculating the pooled results.

## Results

### Literature selection

**Fig. 1:**
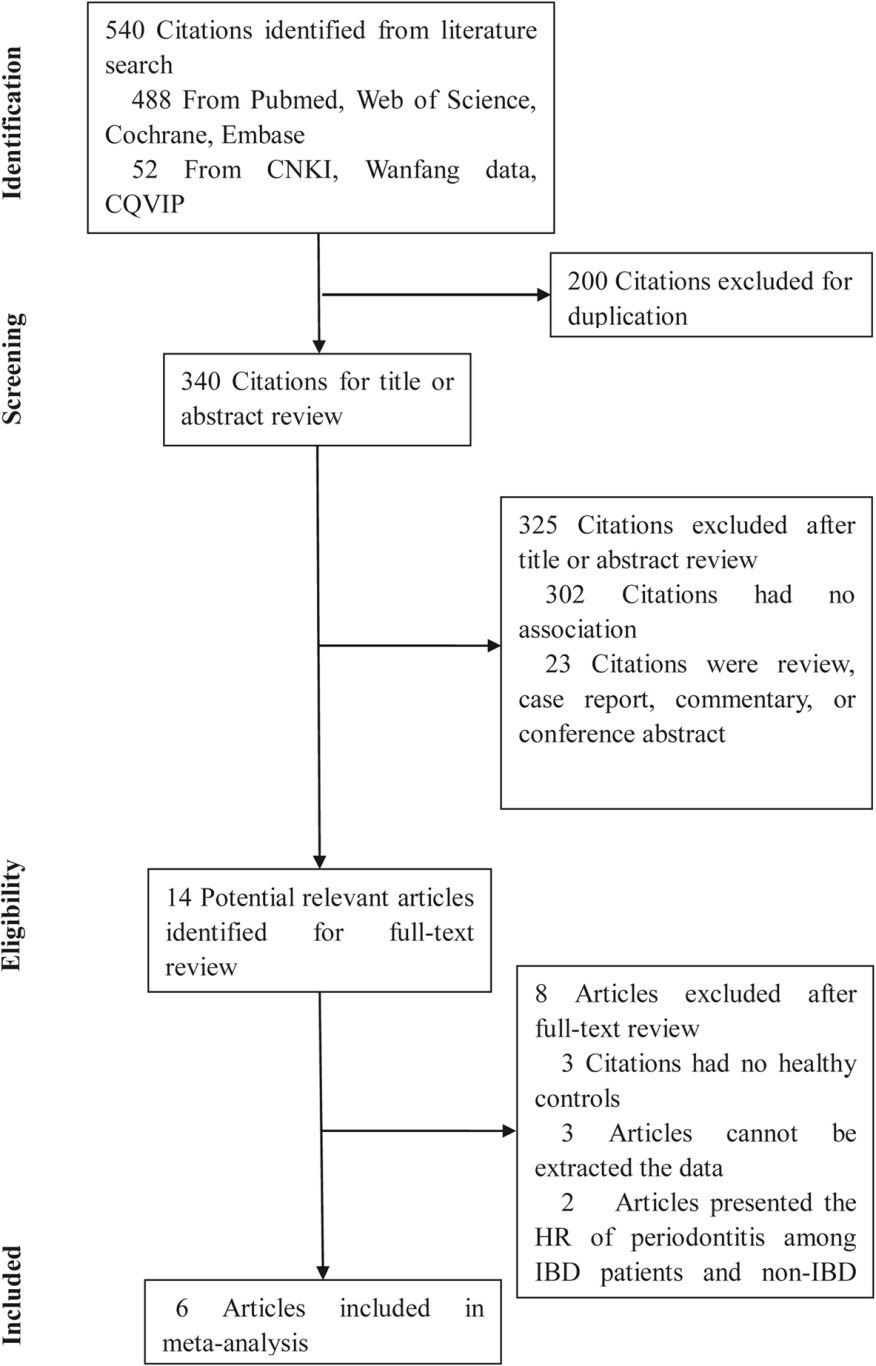
PRISMA flowchart: selection process of studies and results of the literature search for meta-analysis

The literature search distilled 540 citations from databases of Web of Science, Pubmed, Cochrane, Embase, CNKI, Wangfang and CQVIP. After 200 duplications were excluded, 340 studies remained for further consideration. Of these, 325 citations were excluded after screening the titles or abstracts (Fig. 1). On this screening, 302 citations were irrelevant, 23 citations were reviews, laboratory experimental investigations, case reports, or conference abstracts which were all excluded. After this screening, 14 articles were chosen for whole text review. Among these, three articles had no controls, another three articles presented no primary data and two articles presented the hazard ratio (HR) of periodontitis among IBD patients and non-IBD participants and these eight were excluded. Six studies [25–30] were included for final meta-analyses.

### Study characteristics

Characteristics of the included studies in the meta-analysis were shown in Table 1. All studies were published between 2003 and 2015. The participants were 599 IBD patients and 448 controls. Two studies were conducted in Greece and the other four studies were conducted in Germany, Switzerland, Brazil and Jordan respectively. Adjusted odds ratios (*OR*s) and the corresponding 95% *CI* between IBD and periodontitis were available in three of the six case control studies. The estimates were calculated using the numbers of IBD cases (with periodontitis or not) and controls (with periodontitis or not) in the other three studies. All studies had methodological quality with at least five scores according to the NOS.

**Table 1:** Characteristics of included studies in the meta-analysis

Author (Year)	Location	Study design	Matched variables	Participants (M/F, age)	IBD (M/F)	Periodontal measures	Pharmacological treatments	Smoking	Adjusted variables	NOS
Groessner-Schreiber, et al. (2006)	Germany	Matched case–control study	Age; sex; smoking status	IBD:62 (24/38, 38.4) Control:59 (24/35, 38.2)	CD:46 (18/28) UC: 16 (4/12)	PI; BOP; PPD; CAL ≥ 4 mm; CAL ≥ 5 mm	Corticosteroids; immunosuppressants; aminosalicylate; anti TNF Antibiotics	IBD nonsmokers = 34 (55%); smokers = 25; ex-smokers = 3 (5%) Control nonsmokers =29 (49%); smokers = 24; ex-smokers = 6 (10%)	NA	7
Zervou, et al. (2007)	Greece	Matched case–control study	Age; sex	IBD:30 Control:47	CD: 15 UC: 15	Periodontitis	Mesalazine; aziathioprine	OR (95%CI) nonsmokers = 1.00; smokers = 0.99 (0.11–0.63); ex-smokers = 0.61 (0.06–6.29)	Sex; smoking habit; activity duration of disease	6
Brito, et al. (2008)	Brazil	Matched case–control study	Age	IBD:179 (64/115) Control:74 (24/50, 40.3)	CD:99 (31/68) UC:80 (33/47)	BOP; PPD; CAL ≥ 3 mm	Aminosalicylates; immunomodulaors; corticosteroidds; antibiotics; anti TNF-α	CD smokers = 12 (12.1%); nonsmokers = 63 (63.3%); ex-smokers = 24 (24.3%). UC smokers = 7 (8.7%); nonsmokers = 38 (47.5%); ex-smokers = 35 (43.8%) Control smokers = 9 (12.2%); nonsmokers = 57 (77%); ex-smokers = 8 (10.8%)	NA	7
Habashneh, et al. (2012)	Jordan	Case–control study	NA	IBD:160 (94/66) Control:100 (62/38)	CD:59 (33/26) UC:101 (61/40)	PI; GI; PPD; CAL; GR; BOP; PPD ≥ 3; PPD ≥ 4; CAL ≥ 3; CAL ≥ 4; CAL ≥ 5	NA	CD nonsmokers = 23; smokers = 31; ex-smokers = 5 UC nonsmokers = 55; smokers = 17; ex-smokers = 29 Control nonsmokers = 44; smokers = 49; ex-smokers = 7	NA	7
Vavricka, et al. (2013)	Switzerland	Matched case–control study	Age	IBD:113 (65/48, 40.6) Control:113 (58/55, 41.7)	CD: 69 (37/32) UC: 44 (28/16)	PPD; CAL; CAL at deepest pocket; BOP; PBI	Systemic steroids; aminosalicylates; Thiopurines; methotrexate; cyclosporine; or tacrolimus; anti-TNF; probiotics; nonsteroidal; antiinflammatory drug	CD nonsmokers = 25; smokers = 21; ex-smokers = 3 UC nonsmokers = 23; smokers = 2; ex-smokers = 19 Control nonsmokers = 71; smokers = 21; ex-smokers = 21	Perianal extraintestinal manifestation; proctitis; teeth brushing≥twice/day; ex-smoking; HBI>10; CD montreal perianal	7
Koutsochristou, et al. (2015)	Greece	Matched case–control study	Age; Sex; social-economic status	IBD:55 (25/30, 12.3) Control:55 (25/30, 12.2)	CD: 36 (18/18)UC: 19 (7/12)	GI; CPI (PPD)	Aminosalicylate; corticosteroids; anti-TNF; immunomodulators	CD nonsmokers = 36; UC nonsmokers = 19; Control nonsmokers = 55	NA	8

### The association of periodontitis prevalence and IBD

**Fig. 2:**
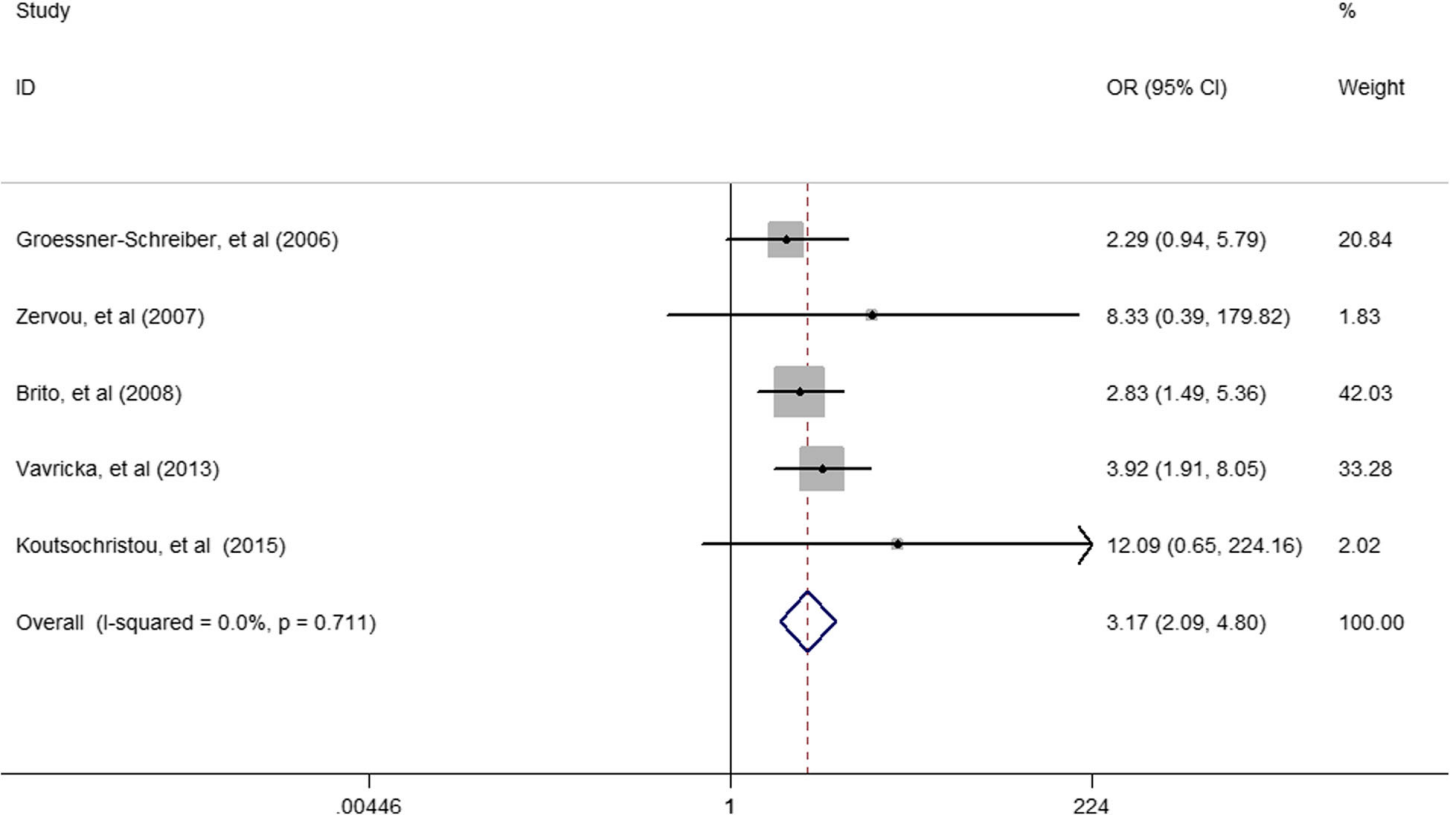
Forest plot displaying the meta-analysis results of association between periodontitis and IBD

**Fig. 3:**
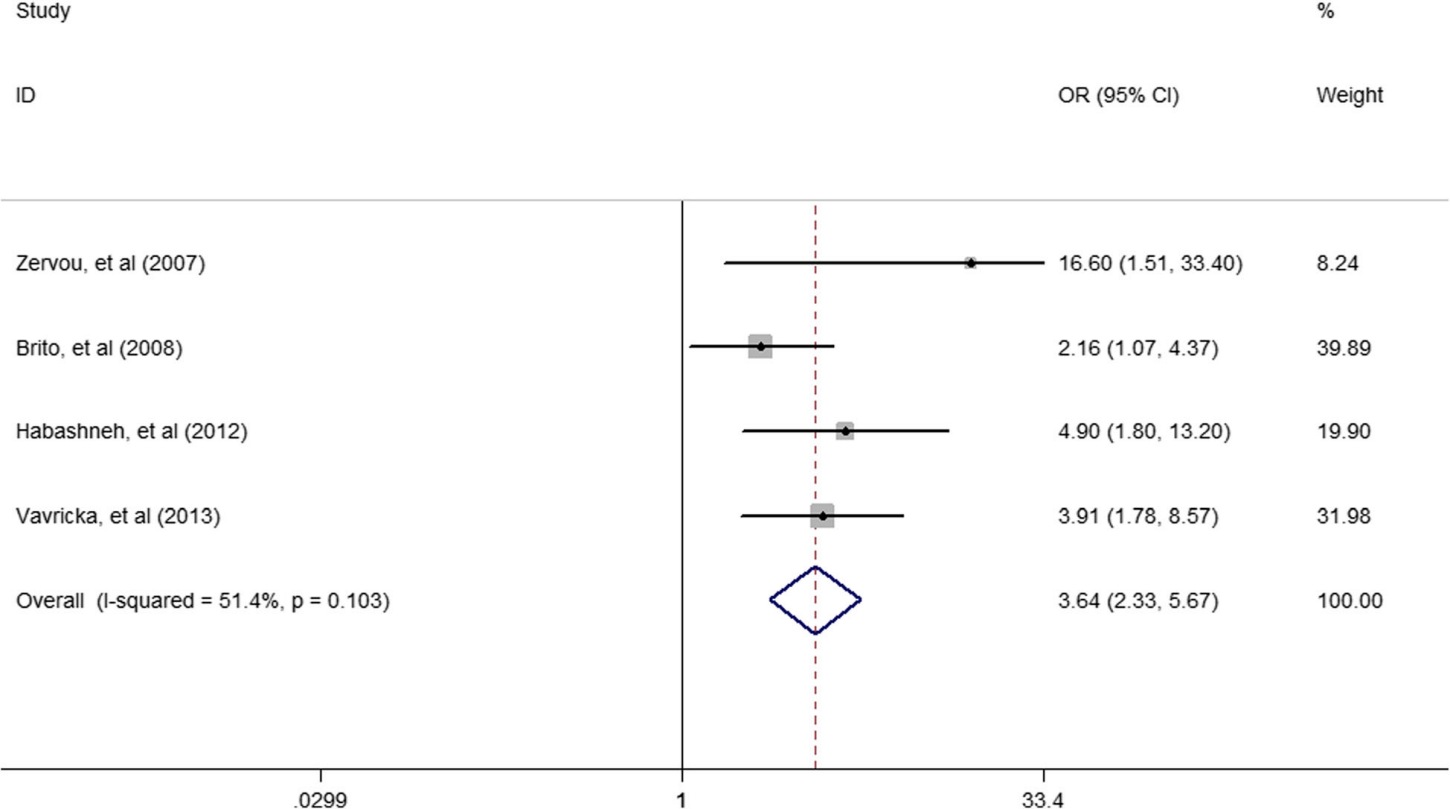
Forest plot displaying the meta-analysis results of association between periodontitis and CD

**Fig. 4:**
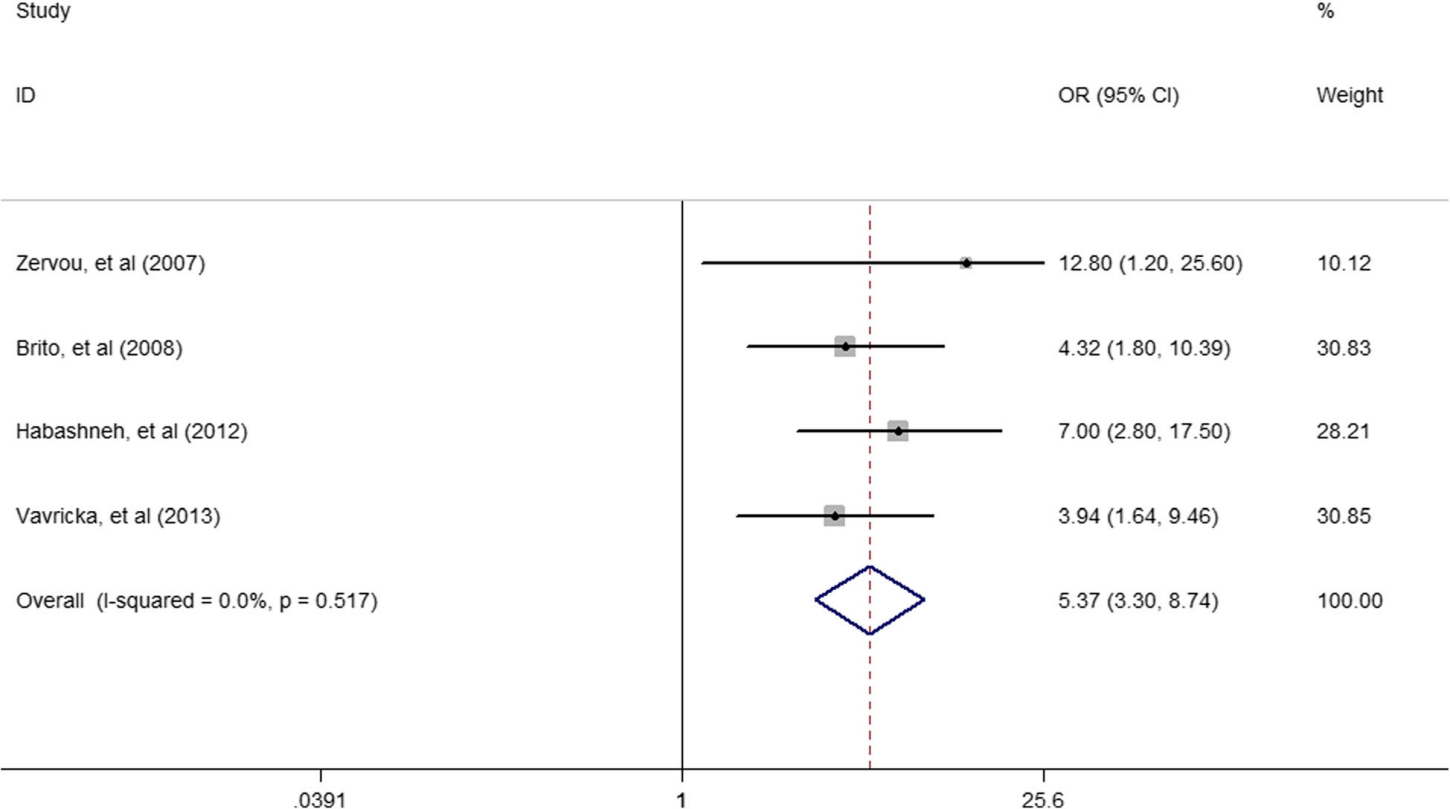
Forest plot displaying the meta-analysis results of association between periodontitis and UC

Among the included studies, the pooled *OR* (95% *CI*) was 3.17 (2.09–4.8) for the association of periodontitis and IBD (Fig. 2). The pooled *OR* (95% *CI*) was 3.64(2.33–5.67) for the association of periodontitis and CD (Fig. 3). The pooled *OR* (95% *CI*) was 5.37 (3.30–8.74) for the association of periodontitis and UC (Fig. 4).

### IBD and measures of periodontitis

Two of the six studies reported oral PI [25, 28]. Four of the studies reported BOP [25, 27–29] and two on GI [28, 30]. Five studies provided data on PPD and four on CAL [25, 27–29].

### Publication bias

**Fig. 5:**
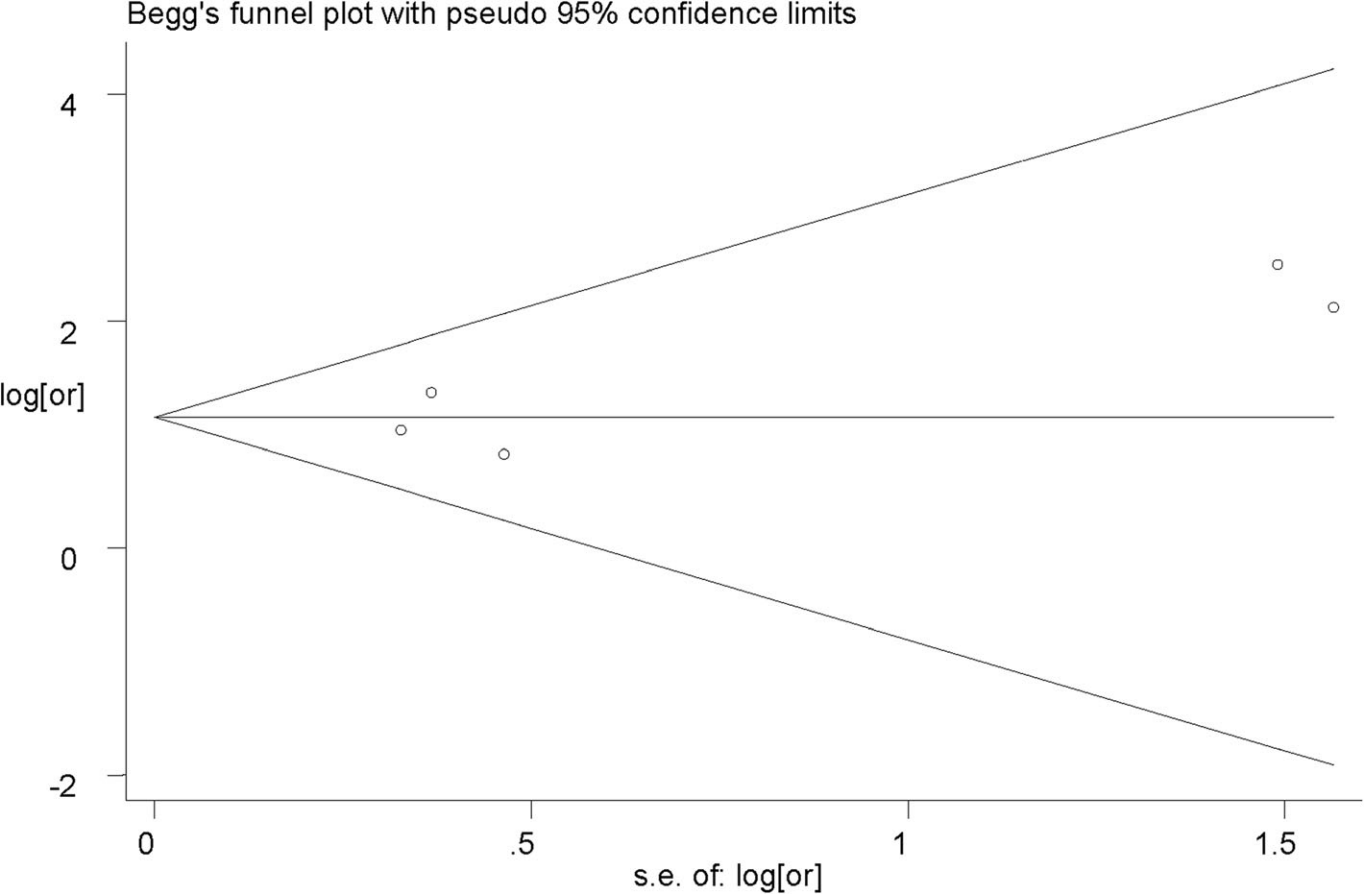
Funnel plot for publication bias regarding the association between periodontitis and IBD

No publication bias was observed (Begg’s test, z = 0.49, and *P* = 0.624; Egger’s test, t = 1.54, and *P* = 0.221). Visual inspection of the funnel plot illustrated a nearly symmetrical distribution, revealing the absence of publication bias (Fig. 5).

### Sensitivity analysis

**Fig. 6:**
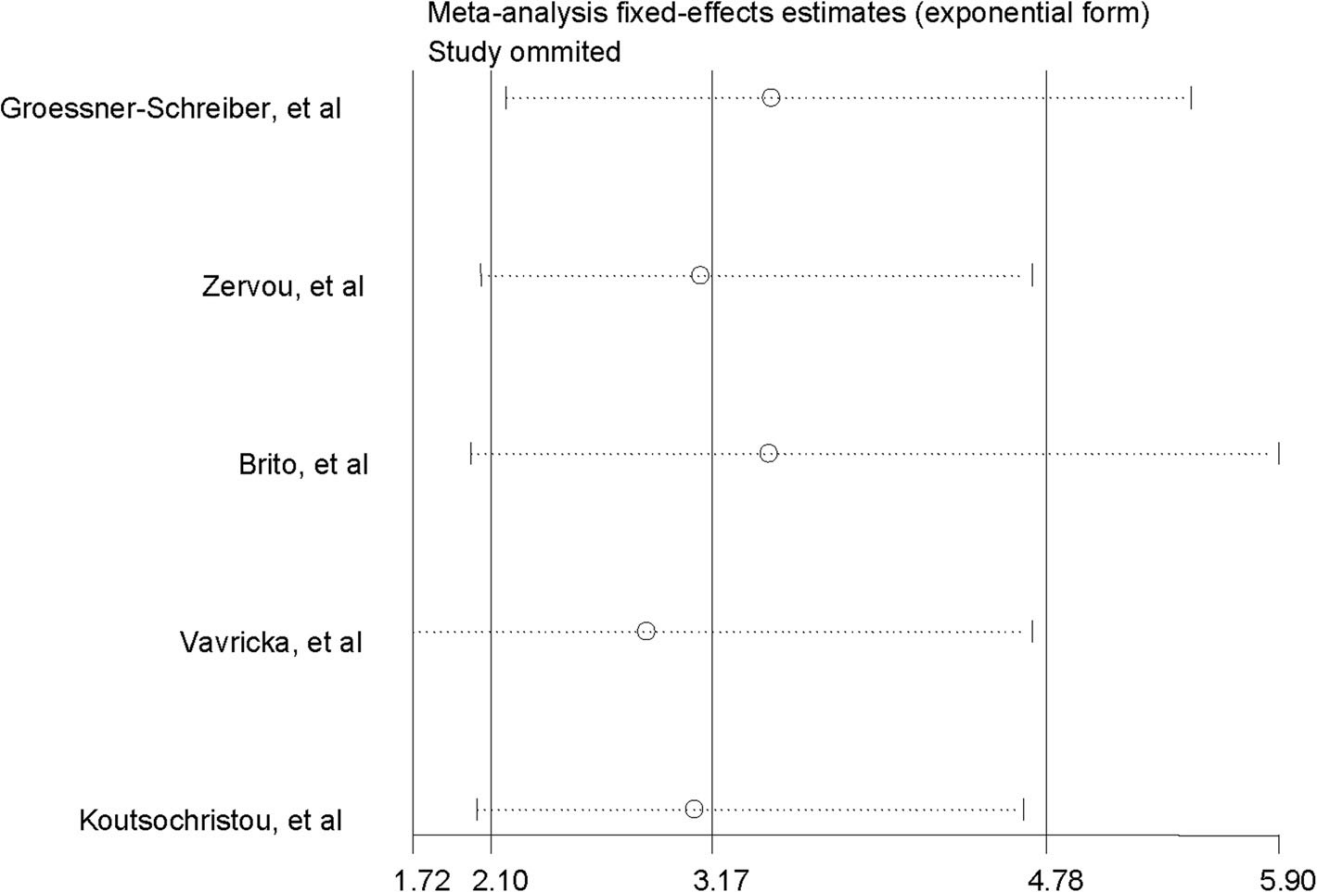
Sensitivity analyses by omitting individual study

The sensitivity analysis showed that the estimates did not vary significantly ranging from 2.85 (95% *CI*: 1.72–4.71) to 3.45 (95% *CI*: 2.18–5.48) when omitting other studies one by one. Generally, the result revealed that no individual study carried enough weight to significantly affect the pooled performance (Fig. 6).

## Discussion

In the present meta-analysis, available evidence was summarized to help clarify the association of periodontitis and IBD. Overall, the results showed a positive association of periodontitis with IBD, CD and UC. According to the results, periodontitis was associated with a higher risk of IBD with low heterogeneity. Sensitivity analysis revealed the robustness of our results and the absence of publication bias added the validity of our results.

Accordingly, periodontitis may have an inverse relationship with IBD with different periodontal tissue destruction. Both CD and UC patients showed deeper pockets compared with the controls in the study of Brito, et al [29]. This is in contrast with Grossner-Schreiber, et al [25], who found deeper pockets in the control group compared with patients with IBD. Most notably, the average CAL was the most important predictive factor for site progression. More sites with CAL were shown in patients with IBD than health controls by Brito, et al [29] and Grossner-Schreiber, et al [25], which highlights the increased risk for periodontal tissue loss among these patients. However, varying extent of periodontal destruction was reflected in UC and CD patients. Compared with UC, CD patients were less vulnerable to CAL and developing sites with CAL ≥ 3 mm. This may indicate a potential difference in the pathophysiology of these two diseases, involving the T helper (Th) cell differentiation. UC is considered to be Th2 disease, while CD has characteristics of a Th1 disease. Another possible explanation for this difference might be due to patients with CD taking markedly more immunomodulators than those with UC [29].

The observational studies on the the association between periodontitis and IBD are revealing, but it could be interfered by several confounding factors, such as gender, smoking, and medications. Thus, it is necessary to rely on properly adjusted risk estimates. One of the primary drivers of periodontitis is smoking, as proposed by the included studies. It has been shown that those who were ex-smokers and those who were having IBD were prevalent for periodontitis. In patients with CD, ex-smoking and clinical activity were significant risk factors for periodontitis [27]. But it seems to play a protective role in UC, with a decrease in the expression of proinflammatory Th1/Th17 cytokines in colon [31]. In addition, in IBD subgroups compared with healthy controls, the existence of perianal extraintestinal manifestations in IBD and proctitis in patients with UC were risk factors for periodontitis [27]. Furthermore, it is noteworthy that patients with IBD taking immunomodulators had a higher mean value of GI [30] and increased needs of periodontal treatment. The drug species applied to the treatment of IBD can lead to alterations on periodontal tissues due to the direct toxic effects, as well as indirect immunodepression effects with developing opportunistic infections [32]. However, in a retrospective cohort study, Chi, et al. [33] expounded an increased HR for subsequent periodontitis among CD patients when compared to matched controls, where treatment of CD showed protection against periodontitis due to the protective effect of some pharmaceuticals.

Although the etiology of IBD is still unclear, it has been hypothesized that IBD is mediated by chronic inflammation triggered by an environmental stimulus in a genetically primed individual [9, 20]. Periodontitis is an inflammatory response caused by the stimulation of colonized Gram-negative bacteria [9, 20]. Microbiological impact has been suggested as a potential factor accountable for the altered predisposition to periodontitis in IBD patients. Van Dyke et al. [34] reported a microflora composed of Gram-negative bacteria that was consistent with the genus Wolinell in a periodontal microflora of IBD patients. Brito et al. [15] showed that a high prevalence of *Treponema denticola* and other bacteria, in connection with opportunistic infections in subgingival sites, were found in IBD patients. The severity of periodontitis might be attributable to the crucial microbe-host interaction. Another linkage between periodontitis and IBD is related to immune-inflammatory response. Specifically, a possible role for G protein-coupled receptor 30 (GPR30) and tumor necrosis factor-α (TNF-α) has been implicated [35–38]. The level of TNF-α is elevated in the gastrointestinal tract of CD patients, as well as in the gingival crevicular fluid of periodontitis patients. GPR30 mRNA and protein expression were detectable in the colonic tissues of IBD patients and may play a role in the intestinal inflammatory balance [36].

At present, our understanding of the mechanism that oral bacteria may contribute to the development of IBD is still evolving. When bacteria find a home in the dental plaque, local pro-inflammatory cytokines produced by the monocytes and macrophages activated by bacteria and their products might enter the systemic circulation [39]. Focusing on the oral bacteria, so as to initiating new ideas for the treatment of IBD. Treating both local oral and systemic inflammation would probably come under the spotlight for the optimal therapeutic strategies.

While a significant association was found between periodontitis and IBD, there were some limitations in this meta-analysis. First, all the studies included were English publications, leading to a possible language bias. Second, regional, ethnic, age, and diagnostic criteria for periodontitis may also be the sources of heterogeneity, but no further subgroup analysis was performed due to the limited number of studies and subjects. Third, not all six studies included here presented the adjusted estimates between periodontitis and IBD. As a result, potential confounding factors could lead to some bias in the association between periodontitis and IBD. Last but not least, the six studies included here were all case-control studies and no prospective large-scale cohort study has been published. The current data cannot be used to establish a cause-and-effect relationship between IBD and periodontitis. Further intervention studies are needed in order to establish such a causal relationship.

## Conclusions

Collectively, within the limitations of this analysis, result of the current data revealed that periodontitis i1s positively associated with IBD. Future mechanistic studies are necessary to elucidate the potential relationship between periodontitis and IBD.

## Data Availability

All data generated or analyzed during the present study are included in this published article.

## Abbreviations

BOP: Bleeding on probing
CAL: Clinical attachment loss
CD: Crohn’s disease
CI: Confidence interval
CPI: Community periodontal index
EIMs: Extraintestinal manifestations
Embase: Excerpta Medica Database
GI: Gingival index
GR: Gingival recession
IBD: Inflammatory bowel disease
IECs: Intestinal epithelial cells
NOS: Newcastle-Ottawa Scale
ORs: Odd ratios
PBI: Papilla bleeding index
PI: Plaque index
PPD: Probing pocket depth
PRISMA: Preferred reporting items for systematic reviews and meta-analyses
Th: T helper
UC: Ulcerative colitis
